# Moving Minds: How Physical Activity Shapes Motivation and Self-Concept in School Children

**DOI:** 10.3390/bs15050629

**Published:** 2025-05-05

**Authors:** Slobodan Pavlović, Vladan Pelemiš, Marko Badrić, Dalibor Stević, Nebojša Mitrović

**Affiliations:** 1 Faculty of Education, University of Kragujevac, 31000 Užice, Serbia; slobodan.b.pavlovic@gmail.com; 2 Faculty of Education, University of Belgrade, 11000 Belgrade, Serbia; 3 Faculty of Teacher Education, University of Zagreb, 10000 Zagreb, Croatia; marko.badric@gmail.com; 4 Faculty of Education, University of East Sarajevo, 76300 Bijeljina, Bosnia and Herzegovina; dalibor.stevic@pfb.ues.rs.ba (D.S.); nebojsa.mitrovic@pfb.ues.rs.ba (N.M.)

**Keywords:** physical activity level, behavioral motivation, physical self-concept, psychological well-being, school children

## Abstract

Background: This study aimed to investigate the differences in motivation and physical self-concept among pupils based on their level of physical activity during physical education classes. Methods: The research encompassed 398 pupils (aged 10 ± 0.3 years), including 211 boys and 187 girls, divided into three groups according to their level of physical activity during class (Group A—low; Group B—medium; Group C—high). The modified Self-Regulation Questionnaire was used to evaluate pupils’ motivational orientations, while the corresponding subscales of the Self-Perception Profile for Children (SPPC) were employed to measure physical self-concept. Pupils’ physical activity (measured in steps and intensity) during physical education classes was assessed using CoachGear pedometers and Suunto Memory Belt heart rate monitors. Results: The findings revealed significant differences between the groups of pupils of both genders concerning their physical activity levels during early school years. Notably, these differences were more pronounced in boys than in girls. Additionally, it was observed that less active pupils generally exhibited lower levels of motivation as well as lower physical self-concept scores. Conclusion: Pupils with higher levels of physical activity during physical education classes demonstrated greater motivation and a more positive physical self-concept, highlighting the crucial role of engagement in fostering both psychological and physical development. These findings underscore the need for well-structured and engaging physical education programs that support active participation and enhance pupils’ overall well-being.

## 1. Introduction

The process of maintaining high-quality physical education classes, as prescribed by the curriculum, encounters numerous challenges in daily practice. On one hand, these challenges arise from insufficient material resources for conducting lessons, while on the other hand, they are closely tied to the quality and structure of the lessons themselves (Zavod za Vrednovanje Kvaliteta Obrazovanja i Vaspitanja, 2008).

An essential prerequisite for physical education to effectively promote healthy development and prepare pupils for an active lifestyle is their full engagement and participation during classes. Since physical education is primarily based on pupils’ motor activities, which are essential for achieving specific instructional objectives, it is crucial that pupils are appropriately engaged. This pertains mainly to the volume and intensity of their motor activity. Current recommendations suggest that moderate to vigorous physical activity during physical education classes should constitute 50% to 60% of instructional time, or the total duration of the class (USDHHS, 2000). However, despite these recommendations, student activity levels in physical education classes remain consistently low in routine teaching practice (Pavlović et al., 2017a; Santos Silva et al., 2019). Observations of physical education classes in over a thousand schools reveal that, on average, student activity reaches only 37% of the instructional time (McKenzie, 2015). The issue of inadequate student activity during physical education classes has been a long-standing challenge in the field. Even in earlier studies (Stanojević, 1961), it was noted that the average duration of physical education classes was approximately 38 min. Of this, only 30 min were dedicated to exercise, with pupils actively participating for just 9 min. Similarly, an older study conducted in Germany found that the class duration was around 35 min, which also represented a notable deviation from the standard class duration (Hoffmann, 1976).

Globally, insufficient physical activity among primary school pupils has been observed, with a study in the United States revealing that pupils were active for only 8.6% of the total class time, and this activity was of moderate intensity, significantly below the 50% recommended by national education guidelines (Simons-Morton et al., 1994). Additionally, more recent studies in the United States continue to highlight the issue of low levels of student activity in schools, with only 13.7% of pupils being engaged in moderate to vigorous physical activity during physical education classes. This indicates a persistent issue that requires comprehensive measures to address within the physical education curriculum (Gill et al., 2019).

Regarding the level of physical activity, both boys and girls exhibit similar levels of activity during physical education classes, though it has been observed that the overall intensity of the lessons is generally low, with girls engaging in physical activity at a lower intensity. This may be due to the content of physical education programs in primary schools, which primarily focus on training and the basic development of motor skills. As a result, children, during periods of rapid growth, are often deprived of more intense physical activity (Đokić, 2014; Pelemiš et al., 2019). Previous studies have indicated that the effective exercise time during physical education classes averages around 15 min (Petrović, 2010; Božović, 2011). In another study, where physical activity was tracked using the SOFIT observational tool, the average active exercise time for pupils was 17 min and 6 s (Marković et al., 2012). This suggests that the engagement period is insufficient to achieve a satisfactory level of instruction and the desired outcomes.

Several personal, behavioral, social, and other factors contribute to the variations in physical activity levels among children and adolescents both in their free time and during physical education classes (Pathare et al., 2016). Among personal factors, motivation and physical self-concept have been identified as significant contributors to pupils’ physical activity during physical education lessons. Motivation to participate in physical education plays a key role in the effective execution of lessons and the annual curriculum. The strength and direction of motivation for physical education vary widely. For some pupils, physical education is the most enjoyable part of the school day, while for others, it is a major stressor and a reason for school absenteeism (Biddle, 2001). Motivational sources can vary, including teachers (Ntoumanis & Standage, 2009), peers (Slingerland et al., 2014), and others. A key aspect of self-determination theory is the differentiated approach to motivation.

Specifically, individuals not only differ in their level of motivation (i.e., how much motivation) but also in the orientation of that motivation (i.e., which type of motivation) (Ryan & Deci, 2000). Self-determination theory categorizes types of motivation based on the degree of autonomy or self-determination, where the most autonomous forms lead to the most positive outcomes. When intrinsically motivated, individuals participate in an activity solely for the satisfaction, challenge, and enjoyment inherent to the activity itself. Intrinsic motivation is considered the ideal for self-determined activity, as participation is voluntary, internally driven, and regulated (internal locus of causality), supported by positive feelings of interest and fun (Ryan & Deci, 2002). The more intrinsically motivated an individual is, the more self-determined their behavior becomes, which is fundamental to psychological health and well-being.

Physical self-concept plays a crucial role in student engagement in physical education and their overall experience of the class (Navarro-Paton et al., 2020). According to Susan Harter’s Theory of Competence Motivation (Harter, 1985), the central goal of successful behavior is the sense of competence. In the context of physical activity, this suggests that if individuals view themselves as physically competent, they are more likely to engage in physical activities, and vice versa. The model posits that actual competence precedes perceived competence, with perceived competence having a more immediate influence on actual competence. Gender differences in physical self-concept have been consistently observed across various age groups in the existing research. Females, in particular, tend to have less favorable perceptions regarding their physical appearance and sports competence (Tubić et al., 2012).

For overweight and less skilled children, positive self-assessment of sports competence may be negatively affected during physical education classes. As a result, these pupils often prefer less active roles in class, such as playing as goalkeepers in football or handball. In physical education, the role of perceived competence is critical, especially since some pupils may lack prior experience in many sports activities (Pavlović, 2017). Pupils who have had positive experiences and who perceive themselves as physically competent are more likely to find physical education engaging and enjoyable, and they are more inclined to participate in physical education to further develop their sports skills (i.e., exhibiting self-determined motivation).

This study aims to explore what happens to the motivation and physical self-concept of younger school-aged children following an assessment of their level of physical activity in physical education classes.

The goal of this research will be to examine the differences in student motivation and physical self-concept based on the level of their physical activity during physical education classes.

## 2. Materials and Methods

The research sample consisted of 398 ten-year-old schoolchildren (M = 10 ± 0.3 years), comprising 211 boys and 187 girls. Based on the measured levels of physical activity during their physical education classes, the participants were stratified into three activity-based groups: low (Group A), moderate (Group B), and high activity (Group C). All children regularly participated in school-based physical education sessions held thrice weekly, aligned with the official national curriculum. The data collection took place across three consecutive spring months (March to May 2024), consistent with a previous methodology (Pelemiš et al., 2024).

Prior to participation, both school personnel and parents were briefed on the study’s aims and procedures. Parents provided written informed consent, in line with the ethical standards for research involving minors, including adherence to the World Medical Association (2013). The research protocol received institutional approval from the Faculty of Education, University of Kragujevac (approval No. 59/032024, issued 5 March 2024) (Pelemiš et al., 2024).

To evaluate the motivational orientations of the pupils, a modified version of the Self-Regulation Questionnaire (Ryan & Connell, 1989) is utilized, adapted by Goudas and colleagues (Goudas et al., 1994) to specifically assess motivation in physical education. This questionnaire has been extensively applied in school populations and is considered appropriate for this research (Murcia et al., 2009). Pupils complete the questionnaire in the classroom after receiving instructions on how to fill it out. The questionnaire is divided into five subscales, each containing three or four items, with the items alternating between subscales. The amotivation subscale includes three items: (1) I really don’t understand why I do physical education; (2) I don’t see the point of physical education classes; (3) I genuinely feel like I’m wasting my time in physical education. The external regulation subscale consists of four items: (1) Because I’ll face consequences if I don’t participate in physical education; (2) because it is expected of me; (3) I do physical education so the teacher won’t be upset with me; (4) because everyone else has to do it. The introjected regulation subscale contains four items: (1) Because I want the physical education teacher to think I’m a good student; (2) because I would feel bad about myself if I didn’t participate in physical education; (3) I would be embarrassed if I didn’t participate; (4) because I would feel guilty if I didn’t do physical education. The identified regulation subscale includes four items: (1) Because I want to learn various sports skills; (2) because it’s important for me to be successful in physical education; (3) because I want to improve in physical education; (4) because I believe physical education is good for my health.

The intrinsic motivation subscale consists of three items: (1) Because I enjoy learning new sports skills; (2) because I find it interesting and exciting to exercise in physical education class; (3) because exercising in physical education class is enjoyable. Respondents express their level of agreement with these statements using a five-point Likert scale: (5) strongly agree, (4) mostly agree, (3) not sure, (2) mostly disagree, and (1) strongly disagree. A separate score is calculated for each subscale. This score is derived by summing the responses for all items within the subscale and dividing the total by the number of items. All items (statements) are framed in the same direction, meaning that higher agreement with a statement reflects a stronger presence of the corresponding motivation type. To evaluate physical self-concept, the relevant subscales from the Self-perception Profile for Children (SPPC—Harter, 1985, 2012) are used. The SPPC is a well-validated tool with proven psychometric properties and has been extensively applied in similar research (Kolovelonis et al., 2013), making it an appropriate choice for this study. This instrument is designed to assess five distinct areas of children’s self-evaluation, along with overall self-evaluation (a total of six subscales). The subscales used to evaluate physical self-concept (Physical Appearance, Sport Competence) each contain six items (statements). Each statement is structured as a two-part sentence, with one part addressing the child’s competent behavior and the other reflecting incompetence (e.g., “Some children are very good at all types of sports, while others feel they are not very good at sports”). All tests used show high reliability coefficients of α = 0.89 to 0.98, and normal data distributions (KS = 0.03 to 0.85).

The respondent is initially asked to choose whether the first or second part of the sentence better describes them, and then to evaluate whether the selected part fully or partially applies to them. The score for each subscale is calculated as the arithmetic mean of the respondent’s answers to the individual statements, where 1 represents the lowest level of competence in the observed domain, and 4 represents the highest.

Pupils’ physical activity during PE classes—both in terms of quantity (step count) and intensity—is objectively monitored using CoachGear pedometers and Suunto Memory Belt heart rate monitors. These devices are selected for their reliability and ease of use, and configured with individualized inputs such as age, height, and body mass. The chest-worn sensor captures real-time heart rate data during exercise, which are later transferred to a computer for analysis (Pelemiš et al., 2024). The heart rate data allows for the classification of physical activity intensity into three zones: light (resting heart rate + up to 25%), moderate (resting heart rate + 25–50%), and vigorous (resting heart rate + above 50%) (Pavlović et al., 2023; Pelemiš et al., 2024). For analytical purposes, the accumulated time spent in the vigorous zone is used as a key indicator of overall activity intensity. When the device is placed on the center of the chest and activated, it records the resting heart rate. Based on these values, exercise intensity zones are determined. Each zone corresponds to a specific level of effort achieved by pupils during physical education classes.

The physical activity of the participants during physical education lessons is measured as part of the regular curriculum. The lessons primarily focus on the kinesiology of complex activities (sports games including basketball, volleyball, and football). Throughout the activities, efforts are made to minimize the teacher’s direct influence on the pupils’ engagement and physical activity levels. Therefore, suitable methods and types of physical exercise are chosen for the physical education classes. Emphasis is placed on movement tasks that allow pupils to independently decide how active they are during the lesson. Since the variables of the volume and intensity of pupils’ physical activity during physical education come from different measurement spaces, standardization is necessary. Groups are determined using the Euclidean distance squared index for both variables, with Ward’s hierarchical cluster analysis method.

Descriptive statistics are calculated for all variables, including the measures of central tendency such as the arithmetic mean (AM) and measures of variability such as the standard deviation (SD). To examine gender differences in overall motor status, multivariate analysis of variance (MANOVA) is used, and any statistically significant differences are tested with univariate analysis of variance (ANOVA). Post hoc tests are used to determine the significant differences between pairs of subject groups for both sexes.

## 3. Results

The entire sample of participants was divided into two groups (211 boys and 187 girls) and further categorized by gender into three groups based on their level of physical activity during physical education lessons. Table 1 and Table 2 show the grouping of participants using Ward’s hierarchical cluster procedure. Differences in motivation and physical self-concept between the groups formed within each gender were assessed using multivariate analysis of variance, with individual variables tested using univariate analysis of variance. The results in Table 1 and Table 2 reveal differences between the groups for both genders, and both tables are summarized and presented as concisely as possible.

As indicated by the results in Table 1, the boys in Group A exhibited the lowest average values for motivation and physical self-concept compared to the other two groups (B and C), with the exception of introjected regulation, which was higher than in Group B but still lower than in Group C. Group B performed better in motivation (except for introjected regulation) and physical self-concept compared to Group A, but showed weaker results compared to Group C. Group C demonstrated superior outcomes in all tested variables compared to both groups A and B for boys.

According to the results presented in Table 2, it can be observed that the girls in Group B displayed the highest levels of amotivation. However, considering that this variable uses an inverse metric, meaning that lower values represent better outcomes, this group is characterized by the poorest performance. Group C of the girls achieved the best results across all tested variables, while Group A demonstrated the weakest performance when compared to the other groups.

According to the results presented in Table 3 and post hoc analysis, the differences between groups in terms of children’s physical activity levels were revealed. In all variables except for the self-determined amotivation, external regulation, and physical appearance variables, differences were observed between the most active group of boys (C) and the less active groups of boys A and B. In terms of grades, the observed differences were greater between boys in groups C and A than between boys in groups B and C.

According to the results shown in Table 4, it can be seen that there are differences between the groups regarding the level of physical activity in girls. In the variables of amotivation, external regulation, and sports competence, no differences were observed between groups A, B, and C. In the more autonomous variables (introjected regulation, identified regulation, and intrinsic motivation), the differences showed statistical significance. Also, in girls, differences were not observed between the groups in the variable of sports competence (they were observed in boys), but they were observed in the variable of physical appearance.

## 4. Discussion

The purpose of this study was to examine the differences in motivation and physical self-concept among younger school-age children, with an average age of 10 ± 1.3 years, in relation to their level of physical activity during physical education classes. The results show significant differences between groups of both genders in terms of physical activity levels among younger pupils, with these differences being more pronounced in boys than in girls, observed separately by gender. It can be assumed that the kinesiology content of the complex activities in physical education classes served as strong enough stimuli to cause these greater differences in boys, but a longitudinal approach would better determine whether sustained activity drives these psychological benefits over time. In terms of motivation, amotivation and external regulation were not predictive variables, while differences between groups were observed regarding the level of physical activity. When it comes to physical self-concept, particularly physical appearance, no differences were found in boys, while girls did not show differences in sports competence. It is noteworthy that differences were observed in four predictive variables for both boys and girls.

As shown in Table 1 and Table 2, Group C from both genders achieved the highest results across all observed variables. A significant finding of this research was that no differences were observed in the amotivation variable between PA levels in boys and girls, even though differences were anticipated, particularly in favor of Group A (Table 3 and Table 4), the least active group. Analysis of the results revealed that Group A had the lowest scores on self-determined motivational achievements, and higher scores on less self-determined achievements. However, this was not a sufficient reason for the differences in all variables to be statistically significant. A positive aspect of these results was that amotivation scores were at their lowest, indicating that the children did not belong to the group that sees no positive or beneficial aspects to participating in physical education classes. The absence of differences between the groups was also evident in external regulation. Considering the younger school-age participants, it is unlikely to observe any disinterest, boredom, or lethargy towards engaging in physical education classes, regardless of their activity level. This is supported by previous research (Telama, 2009; Pavlović et al., 2017b), which indicates that children at this age naturally require movement, and this need persists through their development until adolescence, where the relationship changes significantly. Motivation, as a continuum with external motivation at one end and internal motivation at the other (Harter, 1985; Pavlović et al., 2023), shows that the participants did not differ in amotivation and external (extrinsic) motivation. But in introjected, identified, and internal (intrinsic) motivation, depending on their physical activity in physical education classes, differences existed. Group C (the most active group) (Table 3 and Table 4) stood out significantly in terms of the results achieved. In real-life contexts, including physical education classes, external and internal motivation models rarely function independently. Instead, physical activity typically integrates elements of both models, highlighting their dynamic interaction (Vallerand, 2007).

The possible explanations for the results of this study may be found in the fact that the participants were third- and fourth-grade elementary school pupils, for whom physical education classes are motivated both by the need for personal, intrinsic satisfaction and the desire for external rewards, such as grades or praise. In the case of introjected regulation, behavior is driven by a sense of obligation to avoid guilt, shame, or to boost the ego and sense of self-worth, for instance, the questionnaire statement “If I didn’t participate in physical education, I would feel guilty about it.” It is also important to acknowledge that grading, as an evaluation of achievement, is commonly applied in these school grades, and pupils were likely aiming for a desired grade, striving to reach that goal through external motivation. Both external and introjected regulations result from a predominantly external perceived locus of control, whereas more advanced forms of extrinsic motivation are based on an internal locus of control. This is reflected in the differences between the groups concerning physical activity levels, where Group C, which achieved the best results, significantly outperformed the other groups in all tested variables. When it comes to the most autonomous type of motivation, intrinsic motivation, participants in Group C from both genders showed the highest scores and significant differences compared to the other groups. Previous studies (Vallerand & Losier, 1999) have shown that in the context of physical activity, high intrinsic motivation and low amotivation are associated with the most adaptive cognitive, emotional, and behavioral outcomes. The participants in Group C were more task-focused, promoting the need for autonomy rather than comparing themselves to others. Focusing on and concentrating on the task positively influenced participation in class for intrinsic reasons (enjoyment and fun), which is also supported by the research (Đorđić & Tubić, 2010). Differences in physical self-concept were evident among participants of both genders, though in distinct variables. Fernández-Bustos et al. (2019) found that gender differences often favor boys. The dominance of Group C, the most active participants, was also apparent here. These differences were reflected in boys’ differing results in the sports competence variable, and for girls in the physical appearance variable. Boys in Group C scored highest in assessing their sports competence. Research by Hagger et al. (2005) has shown that boys tend to have a higher physical self-concept in the domains of sports competence, physical fitness, and physical strength. Similar findings on the perception of sports competence and physical self-concept in favor of boys have been reported by other authors (Asci, 2002; Shapka & Keating, 2005). The boys from Group C, who were the most active during physical education, rated their sports competence significantly higher than those from the other two groups (A and B). This suggests that the greater extent and intensity of physical activity in which the boys participated provided them with the confidence to assess their sports competence more favorably (Jekauc et al., 2017). It is also important to note that the teaching units were focused on complex kinesiology activities that required a higher level of motor skills. This emphasis on motor competencies explains the differences observed in boys in relation to their physical activity levels during physical education classes. Additionally, the social context tends to support self-assessment in the sports domain for boys, as the traditional “masculine role” encourages and highly values boys’ participation in sports, expecting them to be stronger, more active, and focused on developing sports competencies, which is less common for girls (Neisen et al., 2007). For the female participants, sports competence was not a significant factor in explaining differences in the level of physical activity during physical education classes. However, when considering physical appearance, differences were observed among the girls based on their activity levels. These differences appeared to favor the participants in Group C. The findings of this study show that the girls in Group C, who were the most active, rated their physical appearance the highest, significantly higher than the other two groups of girls and the overall sample of boys. However, research by Tubić et al. (2012) suggests that other results may exist. The differences in the self-assessment of physical appearance in relation to physical activity levels can be connected to the contrasting ideals of male and female beauty.

The authors note that a muscular, athletic physique may lead to physical appearance satisfaction in boys, though this may not hold true for girls. Societal pressures, often driven by media, favor the promotion of a model-like female body over a sporty one (Gentile et al., 2009), which should not be prioritized for younger school-aged children. Positive self-perception of physical appearance in girls can act as a protective factor against the pressure to conform to the ideal of extreme thinness (Đorđić & Tubić, 2008). Some studies (Lazarević et al., 2008) have shown a significant difference in physical self-concept between groups of pupils engaged in additional kinesiology treatments, favoring those who participated in more activities beyond physical education. These differences were more pronounced in the female sample compared to the male sample based on their level of engagement. Additionally, since physical appearance is constantly observed by both others and oneself, it can be inferred that the most active girls had the highest self-ratings in terms of physical appearance.

This study has several limitations that should be considered when interpreting the findings. One limitation of this study is its cross-sectional design, which prevents causal inferences regarding the relationship between physical activity, motivation, and physical self-concept. Additionally, the use of pedometers and heart rate monitors, while providing objective measures of activity, may not have fully captured all aspects of engagement and effort during physical education classes. The reliance on self-reported questionnaires for assessing motivation and self-concept introduced the possibility of social desirability bias, particularly in younger children who may have difficulty accurately reflecting on their own perceptions. Furthermore, external factors such as teaching style, curriculum differences, and social influences were not accounted for, which could have impacted the observed differences between groups.

## 5. Conclusions

The results obtained in this study support the majority of findings that highlight differences in motivation and physical self-concept related to pupils’ levels of physical activity. The most active children generally demonstrated the highest motivation, although their physical self-concept showed variations. For boys, sports competence played a more significant role in these differences, while for girls, physical appearance was the more prominent factor. Given these insights, it is crucial to conduct an analysis of how physical education lessons are implemented and planned, as current pupils increasingly express dissatisfaction with traditional exercise methods. In planning and designing physical education curricula, the focus must be on creating a supportive environment that promotes lesson engagement, as this is key to boosting motivation and enhancing self-concept in pupils. Teacher and educator training must be updated in line with society’s evolving expectations for school and preschool physical education. They should be equipped to creatively and effectively encourage student participation in physical education classes to achieve the ultimate goal of fostering healthy child development and preparing children for an active lifestyle.

## Figures and Tables

**Table 1:** Descriptive statistics and differences between the groups of boys.

Variables	A (N = 58) AS	SD	B (N = 75) AS	SD	C (N = 78) AS	SD	*f*	*p*
Amotivation	3.45	1.14	3.03	0.69	2.79	0.44	0.74	0.39
External regulation	3.33	1.02	3.21	0.95	2.77	0.55	0.02	0.89
Introjected regulation	4.33	0.86	4.01	0.59	5.88	0.74	1.75	0.02
Identified regulation	3.14	0.22	3.52	0.74	5,99	0.12	8.05	0.01
Intrinsic motivation	3.66	0.36	3.89	0.45	5.29	0.39	2.76	0.00
Sport competence	2.19	0.88	2.87	0.59	3.86	0.47	8.08	0.01
Physical appearance	2.14	1.01	3.46	0.60	3.73	0.66	2.99	0.08

*F* = 7.89; *P* = 0.01. Abbreviations: A—low PA; B—moderate PA; C—high PA; AS—arithmetic mean; SD—standard deviation; *f*—univariate *F*-test; *p*—level of statistical significance of the univariate *F*-test; *F*—multivariate Wilks’ *F*-test; *P*—statistical significance of the multivariate *F*-test.

**Table 2:** Descriptive statistics and differences between the groups of girls.

Variables	A (N = 56) AS	SD	B (N = 68) AS	SD	C (N = 63) AS	SD	*f*	*p*
Amotivation	3.51	1.44	3.59	1.09	3.33	0.39	0.74	0.39
External regulation	3.39	0.88	3.47	0.23	3.45	0.80	0.02	0.89
Introjected regulation	3.71	0.71	3.81	0.99	4.56	0.29	1.75	0.03
Identified regulation	3.15	0.17	3.52	0.62	4.87	0.09	8.05	0.01
Intrinsic motivation	3.92	0.75	4.01	0.55	5.11	0.13	2.76	0.00
Sport competence	2.67	0.97	2.81	0.57	2.88	0.74	8.08	0.09
Physical appearance	3.12	0.39	3.24	0.69	3.94	0.61	2.99	0.01

*F* = 5.95; *P* = 0.01. Abbreviations: A—low PA; B—moderate PA; C—high PA; AS—arithmetic mean; SD—standard deviation; *f*—univariate *F*-test; *p*—level of statistical significance of the univariate *F*-test; *F*—multivariate Wilks’ *F*-test; *P*—statistical significance of the multivariate *F*-test.

**Table 3:** Difference between low activity (A), moderate activity (B), and high activity levels (C) in boys.

Variables	Group		Differences AS	*p*
	A	B		0.310
		C		0.454
Amotivation	B	A		0.310
		C		0.444
	C	A		0.454
		B		0.444
	A	B		0.085
		C		0.071
External regulation	B	A		0.085
		C		0.065
	C	A		0.071
		B		0.065
	A	B		0101
		C		0.031
Introjected regulation	B	A		0.101
		C		0.042
	C	A		0.031
		B		0.042
	A	B		0.111
		C		0.022
Identified regulation	B	A		0.111
		C		0,049
	C	A		0.022
		B		0.049
	A	B		0.025
		C		0.015
Intrinsic motivation	B	A		0.025
		C		0.028
	C	A		0.015
		B		0.028
	A	B		0.098
		C		0.045
Sport competence	B	A		0.098
		C		0.074
	C	A		0.045
		B		0.074
	A	B		0.290
		C		0.424
Physical appearance	B	A		0.290
		C		0.424
	C	A		0.424
		B		0.424

Abbreviations: A—low physical activity; B—moderate physical activity; C—high physical activity; Differences AS—differences in arithmetic means; *p*—level of statistical significance of arithmetic means.

**Table 4:** Difference between low activity (A), moderate activity (B), and high activity levels (C) in girls.

Variables	Group		Differences AS	*p*
	A	B		0.420
		C		0.574
Amotivation	B	A		0.420
		C		0.564
	C	A		0.574
		B		0.564
	A	B		0.100
		C		0.091
External regulation	B	A		0.100
		C		0.095
	C	A		0.091
		B		0.095
	A	B		0.151
		C		0.041
Introjected regulation	B	A		0.151
		C		0.039
	C	A		0.041
		B		0.039
	A	B		0.122
		C		0.029
Identified regulation	B	A		0.122
		C		0.030
	C	A		0.029
		B		0.030
	A	B		0.050
		C		0.017
Intrinsic motivation	B	A		0.050
		C		0.038
	C	A		0.017
		B		0.038
	A	B		0.215
		C		0.088
Sport competence	B	A		0.215
		C		0.093
	C	A		0.088
		B		0.093
	A	B		0.060
		C		0.036
Physical appearance	B	A		0.060
		C		0.049
	C	A		0.036
		B		0.049

Abbreviations: A—low physical activity; B—moderate physical activity; C—high physical activity; Differences AS—differences in arithmetic means; *p*—level of statistical significance of arithmetic means.

## Data Availability

The data presented in this study are available on request from the corresponding author.
